# Does pain pattern matter? Association of intermittent, constant, predictable and unpredictable knee pain to patient-reported knee symptom acceptability in knee osteoarthritis

**DOI:** 10.1016/j.ocarto.2026.100838

**Published:** 2026-06-05

**Authors:** Gillian A. Hawker, Lisa Carlesso, Ian Stanaitis, Tuhina Neogi, Lauren K. King, Jean W. Liew

**Affiliations:** a Department of Medicine, University of Toronto, Toronto, Ontario, Canada; b Research and Innovation Institute, Women's College Hospital, Toronto, Ontario, Canada; c Institute of Health Policy, Management and Evaluation, Dalla Lana School of Public Health, University of Toronto, Toronto, Ontario, Canada; d School of Rehabilitation Science, Faculty of Health Sciences, McMaster University, Hamilton, Ontario, Canada; e Section of Rheumatology, Department of Medicine, Boston University Chobanian & Avedisian School of Medicine, Boston, MA, USA; f Li Ka Shing Research Institute, St. Michael's Hospital, Unity Health Toronto, Toronto, Ontario, Canada; g Section of Rheumatology, Boston University Chobanian and Avedisian School of Medicine, Boston, MA, USA

**Keywords:** Intermittent and constant osteoarthritis pain (ICOAP) measure, Knee osteoarthritis, Pain predictability, Constant pain, Intermittent pain

## Abstract

**Objective:**

People with knee osteoarthritis (OA) describe intermittent, constant, predictable, and unpredictable pain, but how different pain patterns affect symptom experience is unknown.

**Design:**

This secondary cohort analysis included individuals aged ≥30 years with knee OA referred for TKA consultation in Alberta, Canada. Pre-consult questionnaires assessed socio-demographics, health status, and knee symptoms (Intermittent and Constant Osteoarthritis Pain [ICOAP] measure assesses intermittent and constant pain severity, and frequency of predictable and unpredictable intermittent pain; Patient Acceptable Symptom State [PASS] assesses if current knee symptoms are ‘acceptable’ or ‘unacceptable’). Multivariable robust Poisson regression assessed associations of intermittent and constant pain severity and frequency of predictable and unpredictable intermittent pain with unacceptable knee symptoms. We tested for an interaction between intermittent pain severity and unpredictable pain frequency, adjusting for age, sex, education, comorbidity, and troublesome joints.

**Results:**

Of 2360 participants, 1798 with intermittent pain scores >0 and PASS responses were included. 1293 reported intermittent, constant, predictable and unpredictable knee pain; 1268 reported ‘unacceptable’ symptoms. In bivariate analyses, all pain patterns were associated with risk for unacceptable symptoms. In the fully adjusted model, older age and greater intermittent and constant pain were associated with higher risk of unacceptable symptoms. No significant interaction was observed between intermittent pain severity and unpredictable pain frequency.

**Conclusions:**

Among people with advanced symptomatic knee OA, more than two-thirds reported predictable and unpredictable intermittent and constant knee pain. Associations between pain predictability and symptom state acceptability were explained by greater intermittent and constant pain severity. Further research is warranted to confirm these findings across a broader spectrum of knee OA severity.

## 1 Introduction

Joint pain is the foremost feature of osteoarthritis (OA) and the number of people living with painful OA is on the rise. In 1990, it was estimated that 256 million adults were living with painful OA (4.8%); in 2020, this had swelled to 595 million or 7.6% of the global population [1,2]. Painful OA affects sleep, physical function, mental health, ability to work, quality of life and OA-related health care use [[2], [3], [4], [5]]. The effective management of OA pain is challenging due to the heterogeneity of the OA pain experience, the influence of psychosocial factors, and variable underlying pain mechanisms.

In focus groups conducted under the auspices of an OARSI-OMERACT initiative to better understand the OA pain experience [6], people with symptomatic hip/knee OA described two distinct types of pain – one that was ‘intermittent’ and often severe or intense and a more persistent or ‘constant’ pain or aching. They also reported that their intermittent knee pain might occur predictably ‘after a trigger’, e.g., physical activity, or unpredictably, ‘without warning’ [6]. While they considered all intense OA pain distressing, they indicated that *intense unpredictable intermittent pain* was the most distressing. They could plan for and pre-emptively manage predictable knee pain, but unpredictable pain left them exhausted and was a barrier to activity, including valued social activities [6]. These findings informed the development of the OARSI/OMERACT Intermittent and Constant Osteoarthritis Pain (ICOAP) measure [7], comprised of subscales for intermittent pain (pain that ‘comes and goes’) and constant pain (pain present ‘most of the time’). The reliability, validity and responsiveness of the ICOAP subscales has been demonstrated and ICOAP has been translated into more than a dozen languages [7,8].

Clinical studies using ICOAP have demonstrated the independent contributions of intermittent and constant pain to OA outcomes [[9], [10], [11], [12]]. However, as items assessing intermittent pain ‘predictability’ were developed, psychometrically assessed [13], and incorporated into ICOAP after its original release, the contribution, if any, of intermittent pain predictability to OA outcomes has been less well studied. In a community cohort with symptomatic knee OA, we found that intermittent knee pain that was unpredictable was more strongly correlated than predictable knee pain to participants' OA symptom acceptability [14], consistent with what focus group participants reported. However, the study was underpowered to examine these associations adjusting for the severity of intermittent pain, the presence of concomitant constant pain, or for potential confounders. Thus, it is unclear if intermittent pain predictability contributes independently to and/or moderates the association of intermittent pain severity to the patient's OA pain experience.

To address this gap, in an established cohort of individuals with knee OA referred for consultation with a surgeon regarding primary, elective total knee arthroplasty (TKA), we examined the associations of predictable and unpredictable intermittent pain to the presence of ‘unacceptable’ knee symptoms, adjusting for ICOAP intermittent and constant pain severity and potential confounders. We hypothesized that both intermittent and constant knee pain severity would contribute to knee symptom unacceptability, and that the frequency with which the intermittent pain was unpredictable would moderate the intermittent pain – symptom unacceptability association.

## 2 Methods

### 2.1 Setting and design

This cross-sectional study was nested within the BEST-Knee prospective cohort study [15,16]. Participants were recruited from those referred by a physician for consultation regarding primary elective TKA at two orthopedic centralized intake clinics in Alberta, Canada. Patients aged 30 years or older, able to read and comprehend English were eligible. Individuals with inflammatory arthritis were excluded. To examine age and sex effects in the original study, recruitment strived for similar numbers of males and females aged 30–59, 60–69 and ≥ 70 years.

### 2.2 Assessments

Eligible consenting participants completed a standardized pre-consultation questionnaire that assessed socio-demographics (age, sex, post-secondary education, living circumstances), health status (number of comorbidities, other lower extremity troublesome joints), psychological factors (symptoms of depression [Patient Health Questionnaire depression scale, PHQ-8S [17]] and Pain Catastrophizing Scale, PCS [18]) and knee symptom severity (Western Ontario and McMaster University OA Index, WOMAC, pain subscale [19]; Knee Osteoarthritis Outcome Survey Physical Function Short-form, KOOS-PS [20]; Patient Acceptable Symptom State, PASS [21]; and the ICOAP [7,13]). ICOAP is comprised of intermittent and constant pain subscales (scores 0 [no pain] to 100 [extreme pain]) that assess pain intensity, impact on sleep, the degree to which the pain frustrates and upsets, and impact on quality of life. For each subscale, participants may indicate “*I don't have this type of pain*”. Those that report intermittent pain complete two single items assessing the frequency with which the intermittent pain is predictable and unpredictable on Likert scales from ‘never’ (0) to ‘very often’ (4). PASS asks *“Think about all the ways your knee arthritis has affected you during the last 48 h. If you were to remain for the next few months as you were in the PAST 48 HOURS, would this be unacceptable or acceptable to you?”*. Body mass index, BMI, was calculated using participants' height and weight, obtained from clinic records.

Our **primary outcome** was knee symptom acceptability on PASS (unacceptable – yes/no). **Exposures of interest** were the frequencies of predictable and unpredictable intermittent knee pain and the interaction of unpredictable intermittent pain with ICOAP intermittent pain severity (0–100). **Covariates** were ICOAP constant pain severity (0–100) and biopsychosocial factors previously shown to be associated with worse OA symptoms and perceived OA symptom severity: participant age, sex, receipt of post-secondary education, number of other troublesome joints (0–1, 2, 3, 4+), and number of comorbid conditions (0, 1, 2, 3+).

### 2.3 Statistical analysis

Participant characteristics were summarized as frequencies and proportions or means and standard deviations as appropriate. KOOS-PS scores were reverse coded such that higher scores indicated worse disability. Among participants with intermittent knee pain (non-zero ICOAP subscale scores), we assessed the frequencies with which the intermittent pain occurred predictably and unpredictably and the proportion with concomitant constant knee pain (non-zero score for ICOAP constant subscale). To assess if predictable and unpredictable intermittent pain frequency were associated with intermittent and constant pain severity, we assessed the Spearman correlations between intermittent and constant pain with pain predictability. Based on the distributions of responses, we categorized the prevalence of predictable intermittent pain as 0 ‘never/rarely/sometimes’, 1 ‘often’ or 2 ‘very often’, and the frequency of unpredictable pain as 0 ‘never/rarely’, 1 ‘sometimes’, 2 ‘often’ or 3 ‘very often’.

We used multivariable binomial regression with robust variance estimation to examine the associations between the frequencies of predictable (0–2) and unpredictable (0–3) intermittent knee pain and reporting an unacceptable knee symptom state, before and after adjustment for ICOAP subscale scores and potential confounders. Modelling was performed in a stepwise fashion as follows: In step 1, we assessed the unadjusted risk ratios associated with frequencies of predictable and unpredictable intermittent knee pain, separately, and reporting unacceptable knee symptoms. In step 2, we assessed the risk ratios associated with frequencies of predictable and unpredictable intermittent knee pain, together and adjusted for ICOAP subscale scores. We also tested for an interaction between ICOAP intermittent pain and unpredictable intermittent pain frequency. If the interaction was significant, it was retained in subsequent steps. In step 3, we further adjusted the step 2 model for potential confounders (age, sex, comorbidity, other troublesome joints and post-secondary education). Collinearity of variables was assessed using a Variance Inflation Factor of >4 [22]; none was found. Statistical significance was considered at a two-tailed p value < 0.05. Using logistic regression, we repeated step 3 to assess model discrimination of individuals with versus without an unacceptable knee symptom state (model c-statistic) and model fit (Hosmer-Lemeshow Goodness of Fit statistic p value) [23]. Analyses were performed using SAS Version 9.4 (SAS Institute Inc., Cary, NC).

Ethics approval for the BEST-Knee Study was obtained from Research Ethics Boards (REB) of the University of Alberta (PRO-00051108), University of Calgary (REB 14–1294), and Women's College Hospital (REB 2014-0092) at the University of Toronto. Participants provided written informed consent. This study was reported in accordance with the STROBE (STrengthening the Reporting of OBservational Studies in Epidemiology) guidelines [24].

## 3 Results

### 3.1 Study sample and characteristics overall and by unpredictable intermittent pain frequency

Of 2064 eligible knee OA patients referred for consultation regarding TKA, 1798 (87.1%) with non-zero scores for ICOAP intermittent knee pain and PASS responses were included. Participants’ mean age was 65.6 years (standard deviation [SD] 9.1 years), 1041 (57.9%) were female and 323/1776 (18.2%) were living alone (Table 1). Mean BMI (SD) was 32.7 (6.8) kg/m^2^. Over 70% of participants had at least one comorbid condition and at least two other troublesome joints. Mean PHQ-8 score (SD) for depressive symptoms was 6.1/24 (5.7). Mean WOMAC pain and KOOS-PS scores (SD) were 11.5/20 (3.6) and 56.1/100 (17.6), respectively; 1268 (70.5%) reported their knee symptom state as unacceptable.

**Table 1:** Participant Characteristics Overall and by the Presence of Unpredictable Intermittent Knee Pain (n = 1798). The denominator is shown when the response rate was less than 100%.

Characteristic	Overall N = 1798	Frequency of unpredictable intermittent knee pain			
		Never or rarely N = 129	Sometimes N = 463	Often N = 780	Very often N = 426
**Demographic characteristics**					
Mean age, years (SD)	65.6 (9.1)	66.7 (8.1)	66.3 (8.1)	65.8 (9.4)	63.9 (8.9)
Female sex–n (%)	1041 (57.9)	62 (48.1)	261 (56.4)	461 (59.1)	257 (60.3)
Post-secondary education–n (%)	1010/1776 (56.9)	85/127 (66.9)	285/460 (62.0)	425/769 (55.3)	215/420 (51.2)
Lives alone–n (%)	323/1776 (18.2)	18/127 (14.2)	92/457 (20.1)	142/770 (18.4)	71/422 (16.8)
**Health status**					
Mean BMI (SD)	32.7 (6.8)	31.8 (6.7)	33.2 (7.1)	32.5 (6.8)	32.9 (6.6)
Mean PHQ-8 depression/24 (SD)	6.1 (5.7)	3.4 (4.5)	4.2 (4.3)	6.0 (5.3)	8.8 (6.6)
≥ 3 comorbid conditions–n (%)	242/1784 (13.6)	14/127 (11.0)	47/460 (10.2)	109/774 (14.1)	72/423 (17.0)
≥ 3 other troublesome joints–n (%)	418 (23.3)	16 (12.4)	75 (16.2)	186 (23.9)	141 (33.1)
**Knee symptoms**					
Mean WOMAC pain/20 (SD)	11.5 (3.6)	8.7 (3.9)	9.7 (3.2)	11.8 (3.0)	13.9 (3.2)
Mean KOOS-PS/100 (SD)	56.1 (17.6)	45.6 (18.3)	48.0 (14.4)	56.8 (15.4)	67.3 (18.0)
Unacceptable symptoms–n (%)	1268 (70.5)	59 (45.7)	265 (57.2)	579 (74.2)	365 (85.7)
Mean ICOAP intermittent/100 (SD)	62.3 (19.1)	39.6 (19.8)	51.4 (16.4)	63.6 (13.7)	78.7 (14.9)
Mean ICOAP constant/100 (SD)	51.2 (27.2)	31.5 (27.5)	37.6 (23.7)	53.1 (23.3)	68.6 (25.6)
Predictable pain–n (%)					
Never/rarely/sometimes	288 (16.0)	61 (47.3)	136 (29.4)	76 (9.7)	15 (3.5)
Often	771 (42.9)	40 (31.0)	214 (46.2)	457 (58.6)	60 (14.1)
Very often	739 (41.1)	28 (21.7)	113 (24.4)	247 (31.7)	351 (82.4)

Most participants reported concomitant constant knee pain (1,573, 87.5%). Mean (SD) scores for ICOAP intermittent and constant pain were 62.3/100 (19.1) and 51.2/100 (27.2), respectively (Table 1). Most participants (1,669, 92.8%) reported that their intermittent knee pain occurred both predictably and unpredictably at least ‘sometimes’; 84.0% and 67.1%, respectively, reported predictable and unpredictable intermittent knee pain ‘often’ or ‘very often’ and 62.0% reported both. Those with a higher frequency of unpredictable intermittent knee pain had received less education, had greater comorbidity and other joint complaints, more depressive symptoms, higher pain catastrophizing, and higher (worse) scores for knee pain and disability than those with less frequent unpredictable pain.

### 3.2 Spearman correlations for ICOAP subscales and predictability items

The Spearman correlation coefficients for the ICOAP predictability items was 0.46. For ICOAP intermittent pain, the correlations were 0.53 with predictable pain frequency and 0.59 with unpredictable pain frequency. For ICOAP constant pain, the correlations were 0.34 and 0.48, respectively (p < 0.0001 for all).

### 3.3 Association of ICOAP pain patterns with knee symptom state acceptability: results of binomial regression with robust variance estimation

*Step 1:* For both predictable and unpredictable intermittent knee pain, a higher frequency was associated with a greater risk ratio for reporting unacceptable knee symptoms (Table 2). For predictable intermittent pain, unadjusted risk ratios and 95% confidence intervals (CIs) were 1.29 (1.14–1.45) for those with predictable pain ‘often’ and 1.52 (1.36–1.71) for those with predictable pain ‘very often’ compared to those with predictable pain ‘never’, ‘rarely’ or ‘sometimes’. For unpredictable intermittent pain, the unadjusted risk ratios and 95% CIs were 1.25 (1.02–1.53), 1.62 (1.34–1.97) and 1.87 (1.55–2.27) for those with unpredictable pain ‘sometimes’, ‘often’ or ‘very often’, respectively, compared to those with unpredictable intermittent pain ‘never’ or ‘rarely’.

**Table 2:** Risk ratios and 95% confidence intervals associated with knee pain predictability for reporting an unacceptable knee symptom state unadjusted and adjusted for ICOAP subscale scores and potential confounders.

Independent variable	Step 1: Unadjusted risk ratios (95% CI)	Step 2: Risk ratios (95% CI) adjusted for ICOAP scores	Step 3: Risk ratios (95% CI) adjusted for ICOAP scores and potential Confounders^a^
ICOAP intermittent/100 per unit increase	**–**	**1.02 (1.01–1.03)**	**1.01 (1.01–1.01)**
ICOAP constant/100 per unit increase	**–**	**1.02 (1.02–1.03)**	**1.01 (1.00–1.01)**
Predictable pain frequency–ref = 0			
1	**1.29 (1.14–1.45)**	0.96 (0.86–1.08)	1.01 (0.90–1.14)
2	**1.52 (1.36–1.71)**	0.96 (0.86–1.08)	1.01 (0.89–1.13)
Unpredictable pain frequency–ref = 0			
1	**1.25 (1.02–1.53)**	0.83 (0.50–1.38)	1.05 (0.87–1.27)
2	**1.62 (1.34–1.97)**	0.98 (0.62–1.56)	1.12 (0.93–1.34)
3	**1.87 (1.55–2.27)**	0.84 (0.50–1.40)	1.03 (0.85–1.24)
Multivariable logistic regression:			
Model c-statistic		0.780	0.784
Hosmer-Lemeshow goodness of fit p value		0.12	0.24

The frequency of predictable pain was categorized as 0 (never/rarely/sometimes), 1 (often) or 2 (very often).

The frequency of unpredictable pain was categorized as 0 (never/rarely), 1 (sometimes), 2 (often) or 3 (very often).

Bolding indicates statistical significance at a p value < 0.05.

P values for interactions between ICOAP intermittent scores and frequency of predictable and unpredictable intermittent knee pain were non-significant (p > 0.05 for both).

a Potential confounders were participant age, sex, post-secondary education, number of comorbidities and number of other troublesome joints.

*Step 2:* In the model that included ICOAP intermittent and constant pain scores and frequencies of both predictable and unpredictable pain, the risk ratios associated with predictable and unpredictable pain frequencies were attenuated, becoming non-significant. Further, no interaction was found between ICOAP intermittent pain severity and the frequency of unpredictable intermittent pain (p value for interaction >0.05). Higher scores for ICOAP intermittent and constant pain were associated with greater risk ratios for reporting unacceptable knee symptoms (adjusted risk ratio, 95% CI, per unit increase in score 1.02, 1.01–1.03, and 1.02, 1.02–1.03, respectively).

*Step 3:* Further adjustment for potential confounders did not alter the findings.

*Model discrimination and fit:* The multivariable logistic regression model comprised of ICOAP subscale and predictability scores and potential confounders had a c-statistic of 0.784 and good model fit (p = 0.24).

## 4 Discussion

In a large cohort of individuals with knee OA referred for consultation regarding TKA, this study assessed the associations of knee pain patterns, assessed with the ICOAP questionnaire, with patient-reported knee symptom state acceptability, assessed using PASS. Adjusting for ICOAP intermittent and constant pain severity, neither the frequency of predictable nor unpredictable intermittent knee pain was associated with knee symptom state acceptability. There was also no evidence that the frequency of unpredictable intermittent pain modified the relationship between ICOAP intermittent pain and knee symptom state acceptability. These findings indicate that pain predictability contributes to unacceptable knee symptoms through its effect on intermittent and constant pain severity. However, research is needed to confirm these findings in populations with a wider range of OA pain severity.

The current study did not find that *unpredictable* intermittent knee pain was more distressful to people living with symptomatic knee OA than a similar level of intermittent pain that was *predictable* [6]. Although the unadjusted risk ratio associated with unpredictable pain ‘very often’ was higher that for predictable pain ‘very often’, the confidence intervals for the estimates overlapped. These findings may reflect the lack of variability in ICOAP and predictability responses in this cohort with relatively advanced knee OA given that study participants were recruited at referral for consultation with a surgeon regarding TKA. As noted above, studies are needed that include individuals with and without unpredictable intermittent pain and with and without constant knee pain to further elaborate the relative impact of pain predictability on OA-related quality of life.

Although we did not have information regarding radiographic severity or OA symptom duration, the Spearman correlations across ICOAP pain types indicated that as intermittent knee pain intensifies, the prevalence and severity of predictable and unpredictable intermittent pain, and also constant knee pain increases. These findings are consistent with previous cross-sectional and longitudinal studies that have shown that intermittent knee pain in people with symptomatic knee OA is more prevalent than constant pain, constant pain is associated with worse intermittent pain, and worse intermittent and constant pain are associated with greater structural severity on imaging and higher likelihood of demonstrating features pain sensitization [[9], [10], [11]]. Longitudinal studies are needed to confirm the evolution of OA knee pain with disease progression. The move towards identifying people with symptomatic knee OA at an earlier structural stage [25] presents a terrific opportunity to do so.

The small to moderate Spearman correlations between pain types found in our study (r = 0.34 to 0.59) indicates that predictable intermittent, unpredictable intermittent and constant knee pain are distinct constructs in knee OA, each contributing to patients’ OA pain experience. The etiologies of these different types of OA pain are unknown. Activity-related (i.e., predictable) intermittent joint pain likely represents nociceptive pain arising from changes in the underlying structural joint pathology and the release of inflammatory mediators in cartilage, bone, and synovium [26]. Bone marrow lesions and synovitis/effusion have been associated with knee pain severity and pain fluctuation [[27], [28], [29]]. Inflammatory mediators may also promote peripheral and central sensitization [30], which has caused some to question whether unpredictable intermittent and/or constant pain in knee OA signifies central dysregulation and sensitization. Indeed, higher ICOAP subscales scores have been linked with the presence of diffuse knee pain and symptoms suggesting nociplastic pain [31], while Carlesso et al. showed that greater pain sensitization, based on quantitative sensory testing, was associated with a higher likelihood of having unpredictable intermittent and constant knee pain [11]. The relationship of different patterns of knee pain to nociceptive and nociplastic pain mechanisms warrants further evaluation.

The OA pain experience was assessed using PASS. While PASS has largely been used in OA clinical research as an anchor measure for calculation of ‘minimal important change’ in patient-reported measures of pain and function [32], PASS was selected as it assesses whether the level of knee symptom severity is considered by the patient to be beyond that at which they would consider themselves well or happy with their current state. Supporting this, ICOAP scores had excellent ability to discriminate individuals who reported their knee OA symptoms as acceptable versus unacceptable. Still, our findings may not be generalizable to studies using other measures of the OA pain experience. Further research is warranted.

Strengths of our study include the large sample that enabled consideration of and adjustment for key factors known to confound the relationship between pain and OA outcomes. However, there were also limitations. In addition to those already noted, we did not have information on joint structure from imaging or findings from quantitative sensory testing. Thus, we could not examine the association of pain predictability to structural severity or pain sensitization. We also had no information about the presence of widespread pain or fibromyalgia diagnosis and therefore our results may be biased by unmeasured confounding.

In conclusion, among people with knee OA considering elective TKA, unpredictable intermittent knee pain was not associated with greater risk of reporting an unacceptable knee symptom state after adjusting for intermittent and constant pain severity. However, we strongly recommend further research to confirm or refute these findings in individuals with a broader range of ICOAP scores. Studies are also needed to better understand the mechanisms underlying these types of pain as they may be targets for future intervention.

## Author contributions

All authors have contributed to the following: (1) the conception and design of the study, acquisition of data, or analysis and interpretation of data; (2) drafting the article or revising it critically for important intellectual content; and (3) final approval of the version to be submitted. Gillian Hawker takes responsibility for the integrity of the work from inception to finished article.

## Role of the funding source

The BEST-Knee Study was funded by an Operating Grant from the 10.13039/501100000024Canadian Institutes of Health Research, grant #MOP-312807. Tuhina Neogi was supported by 10.13039/100000002NIH K24070892 and P30 AR072571. Lauren King was supported by an 10.13039/501100000142Arthritis Society Canada Stars Career Development Award (Star-24-0095). Jean Liew was supported by 10.13039/100000002NIH NIAMS K23 AR082938. The funders had no role in the design and conduct of the study; collection, management, analysis and interpretation of the data; preparation, review or approval of the manuscript; or the decision to submit the manuscript for publication.

## Conflict of interest

The authors report no competing interests related to this work.

## References

[1] Ward Z.J., Goldie S.J. (2024). Global burden of disease study 2021 estimates: implications for health policy and research. Lancet.

[2] GBD 2021 Osteoarthritis Collaborators (2023). Global, regional, and national burden of osteoarthritis, 1990-2020 and projections to 2050: a systematic analysis for the global burden of disease study 2021. Lancet Rheumatol..

[3] Power J.D., Badley E.M., French M.R., Wall A.J., Hawker G.A. (2008). Fatigue in osteoarthritis: a qualitative study. BMC Muscoskelet. Disord..

[4] Hawker G.A., French M.R., Waugh E.J., Gignac M.A., Cheung C., Murray B.J. (2010). The multidimensionality of sleep quality and its relationship to fatigue in older adults with painful osteoarthritis. Osteoarthr. Cartil..

[5] Hawker G.A., Gignac M.A., Badley E., Davis A.M., French M.R., Li Y. (2011). A longitudinal study to explain the pain-depression link in older adults with osteoarthritis. Arthritis Care Res..

[6] Hawker G.A., Stewart L., French M.R., Cibere J., Jordan J.M., March L. (2008). Understanding the pain experience in hip and knee osteoarthritis--an OARSI/OMERACT initiative. Osteoarthr. Cartilage.

[7] Hawker G.A., Davis A.M., French M.R., Cibere J., Jordan J.M., March L. (2008). Development and preliminary psychometric testing of a new OA pain measure--an OARSI/OMERACT initiative. Osteoarthr. Cartilage.

[8] Davis A.M., Lohmander L.S., Wong R., Venkataramanan V., Hawker G.A. (2010). Evaluating the responsiveness of the ICOAP following hip or knee replacement. Osteoarthr. Cartilage.

[9] de Lange-Brokaar B.J., Bijsterbosch J., Kornaat P.R., Yusuf E., Ioan-Facsinay A., Zuurmond A.M. (2016). Radiographic progression of knee osteoarthritis is associated with MRI abnormalities in both the patellofemoral and tibiofemoral joint. Osteoarthr. Cartilage.

[10] Philpott H.T., Birmingham T.B., Pinto R., Primeau C.A., Arsenault D., Lanting B.A. (2022). Synovitis is associated with constant pain in knee osteoarthritis: a cross-sectional study of OMERACT knee ultrasound scores. J. Rheumatol..

[11] Carlesso L.C., Law L.F., Wang N., Nevitt M., Lewis C.E., Neogi T. (2022). Association of pain sensitization and conditioned pain modulation to pain patterns in knee osteoarthritis. Arthritis Care Res..

[12] Davison M.J., Ioannidis G., Maly M.R., Adachi J.D., Beattie K.A. (2016). Intermittent and constant pain and physical function or performance in men and women with knee osteoarthritis: data from the osteoarthritis initiative. Clin. Rheumatol..

[13] Hawker G.A., Chui H., Stanaitis I., King L.K., Davis A.M. (2025). The construct validity and responsiveness of measures of the predictability of intermittent knee pain in knee osteoarthritis. Osteoarthr. Cartil. Open.

[14] Liu A., Kendzerska T., Stanaitis I., Hawker G. (2014). The relationship between knee pain characteristics and symptom state acceptability in people with knee osteoarthritis. Osteoarthr. Cartilage.

[15] Hawker G.A., Bohm E., Dunbar M.J., Faris P., Jones C.A., Noseworthy T. (2023). Patient appropriateness for total knee arthroplasty and predicted probability of a good outcome. RMD. Open.

[16] Hawker G.A., Bohm E., Dunbar M.J., Jones C.A., Noseworthy T., Marshall D.A. (2022). The effect of patient age and surgical appropriateness and their influence on surgeon recommendations for primary TKA: a cross-sectional study of 2,037 patients. J. Bone. Joint. Surg. Am..

[17] Kroenke K., Strine T.W., Spitzer R.L., Williams J.B., Berry J.T., Mokdad A.H. (2009). The PHQ-8 as a measure of current depression in the general population. J. Affect. Disord..

[18] Sullivan M.J.L., Bishop S.R., Pivik J. (1995). The pain catastrophizing scale: development and validation. Psychol. Assess..

[19] Bellamy N. (1989). Pain assessment in osteoarthritis: experience with the WOMAC osteoarthritis index. Semin. Arthritis Rheum..

[20] Perruccio A.V., Lohmander L., Canizares M., Tennant A., Hawker G.A., Conaghan P.G. (2008). The development of a short measure of physical function for knee OA KOOS-physical function shortform (KOOS-PS) - an OARSI/OMERACT initiative. Osteoarthr. Cartil..

[21] Tubach F., Ravaud P., Baron G., Falissard B., Logeart I., Bellamy N. (2005). Evaluation of clinically relevant states in patient reported outcomes in knee and hip osteoarthritis: the patient acceptable symptom state. Ann. Rheum. Dis..

[22] Freund R., Wilson W. (1998).

[23] Hosmer D.W., Hosmer T., Le Cessie S., Lemeshow S. (1997). A comparison of goodness-of-fit tests for the logistic regression model. Stat. Med..

[24] von Elm E., Altman D.G., Egger M., Pocock S.J., Gøtzsche P.C., Vandenbroucke J.P. (2008). The strengthening the reporting of observational studies in epidemiology (STROBE) statement: guidelines for reporting observational studies. J. Clin. Epidemiol..

[25] Mahmoudian A., King L.K., Liew J.W., Wang Q., Appleton C.T., Englund M. (2024). Timing is everything: towards classification criteria for early-stage symptomatic knee osteoarthritis. Osteoarthr. Cartilage.

[26] Atukorala I., Pathmeswaran A., Makovey J., Metcalf B., Bennell K.L., March L. (2021). Can pain flares in knee osteoarthritis be predicted?. Scand. J. Rheumatol..

[27] Cross M., Dubouis L., Mangin M., Hunter D.J., March L., Hawker G. (2017). Defining flare in osteoarthritis of the hip and knee: a systematic literature review - OMERACT virtual special interest group. J. Rheumatol..

[28] King L.K., Epstein J., Cross M., Buzzi M., Buttel T., Cembalo S.M. (2021). Endorsement of the domains of knee and hip osteoarthritis (OA) flare: a report from the OMERACT 2020 inaugural virtual consensus vote from the flares in OA working group. Semin. Arthritis Rheum..

[29] Neogi T. (2017). Structural correlates of pain in osteoarthritis. Clin. Exp. Rheumatol..

[30] Binvignat M., Sellam J., Berenbaum F., Felson D.T. (2024). The role of obesity and adipose tissue dysfunction in osteoarthritis pain. Nat. Rev. Rheumatol..

[31] Fitzsimmons M., Carr E., Woodhouse L., Bostick G.P. (2018). Development and persistence of suspected neuropathic pain after total knee arthroplasty in individuals with osteoarthritis. Pharm. Manag. PM R.

[32] Tubach F., Ravaud P., Martin-Mola E., Awada H., Bellamy N., Bombardier C. (2012). Minimum clinically important improvement and patient acceptable symptom state in pain and function in rheumatoid arthritis, ankylosing spondylitis, chronic back pain, hand osteoarthritis, and hip and knee osteoarthritis: results from a prospective multinational study. Arthritis Care Res..

